# Inhibiting bladder tumor growth with a cell penetrating R11 peptide derived from the p53 C-terminus

**DOI:** 10.18632/oncotarget.5622

**Published:** 2015-10-08

**Authors:** Tingting Zhang, Kaijie Wu, Chen Ding, Kangwei Sun, Zhenfeng Guan, Xinyang Wang, Jer-Tsong Hsieh, Dalin He, Jinhai Fan

**Affiliations:** ^1^ Department of Urology, The First Affiliated Hospital of Xi'an Jiaotong University, Xi'an, Shaanxi Province, China; ^2^ Department of Urology, Xiangyang Central Hospital, Hubei University of Arts and Science, Xiangyang, Hubei Province, China; ^3^ Department of Urology, University of Texas Southwestern Medical Center, Dallas, TX, USA

**Keywords:** bladder cancer, cell penetrating peptide, p53 C-terminus, targeted therapy, metastatic tumor

## Abstract

Urothelial carcinoma of the bladder (UCB) is the most common malignancy of the urinary tract, nearly half of which contains a mutation in *TP53* gene. Hence, therapeutic approach by restoring functional p53 protein in cancer cells will be beneficial. Recent studies have demonstrated the inhibition of cancer cell growth by p53 reactivation using a peptide derived from the p53 C-terminus (p53C). However, the outcome of reactivating p53 in controlling bladder cancer development is limited by its efficiency and specificity of peptide delivery, especially in metastatic animal models. Herein, we report that the cell penetrating peptide (polyarginine, R11)-conjugated p53C can exhibit a preferential uptake and growth inhibit of UCB cells expressing either mutant or wild-type *TP53* by the activation of p53-dependent pathway. R11-p53C peptide treatment of preclinical orthotopic and metastatic bladder cancer models significantly decreased the tumor burden and increased the lifespan without a significant cytotoxicity. Based on these results, we believe that R11-p53C peptide has therapeutic potential for primary and metastatic bladder cancer, and R11-mediated transduction may be a useful strategy for the therapeutic delivery of large tumor suppressor molecules to tumor cells *in vitro* and *in vivo*.

## INTRODUCTION

Urothelial carcinoma of the bladder (UCB) is the 9^th^ most common cancer diagnosed worldwide and the most common malignancy of the urinary tract [1]. Among the initial diagnosis of bladder cancers, approximately one third are diagnosed as invasive disease or have metastasis. Epidemiological data demonstrated that more than 50% of human malignancies, including bladder cancer, are associated with the mutations of *TP53* gene [2]. Mutant p53 proteins not only fail to inhibit tumor growth, but also accelerate tumorigenesis and are associated with poor prognosis of patients and resistance to radiotherapy or chemotherapy [3]. Thus, it is imperative to develop new strategies, such as p53-specific molecular targeted therapy, for achieving a better therapeutic efficacy for this form of UCB.

Restoration of wild-type (wt) p53 function is known to induce inhibition of tumor growth through the expression of downstream genes, such as *WAF1/p21/Cip1, Bax* and *Fas/APO-1*, which play important roles in arresting the cell cycle or inducing apoptosis [4]. Previous studies have indicated that peptides derived from the p53 C-terminus (p53C) can restore the specific DNA sequences binding and transactivational function of mutant p53, resulting in p53-dependent apoptosis in tumor cells [5, 6]. However, these compounds are unable to cross the cell membrane due to the lipophilic nature and effective barrier of the biological membranes, which restricts the intracellular delivery of macromolecules with non-polar and larger than 500D in size [7].

Our previous study indicated that the cell penetrating peptides (CPPs) poly(11)-arginine termed R11 exhibited a higher uptake efficiency by different UCB cell lines [8]. Moreover, *in vivo* evaluation of the tissue distribution of R11 in nude mice showed that R11 exhibited an organ-specific uptake in bladder and prostate tissues after intravenous delivery [9–11]. Thus, R11 could be used as a potential delivery vehicle in UCB therapy. In the present study, our results further indicated that the synthetic peptide conjugated with R11 (R11-p53C) was efficiently and preferentially delivered into bladder cancer cells, resulting in the inhibition of tumor growth regardless of the status of *p53*. More interestingly, this inhibition manifested as a high efficiency in both primary and metastatic tumor models without a significant toxicity.

## RESULTS

### Efficient uptake and growth inhibition of R11-p53C in UCB cells

**Figure 1:**
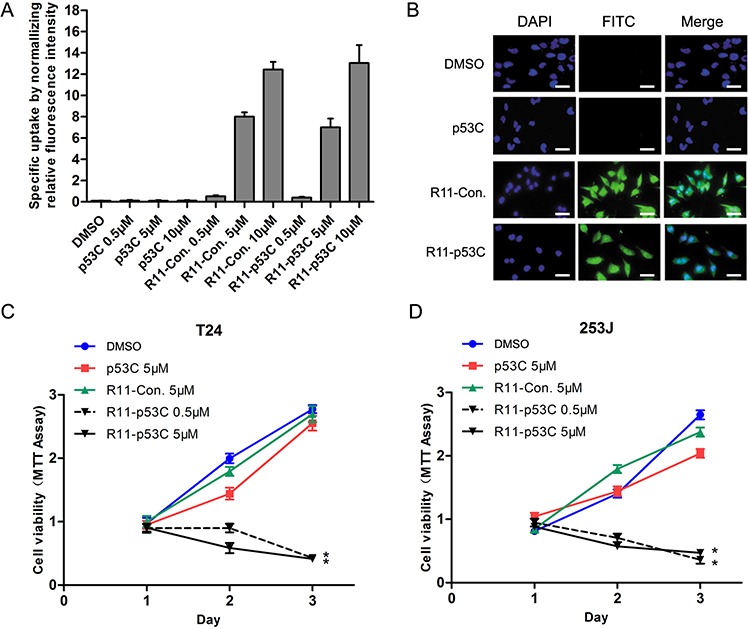
Uptake of R11-p53C and its effects on growth inhibition **A.** The specific uptake of R11-conjugated peptides was determined in T24 cells using fluorometry. **B.** The localization of R11-p53C peptide and its control peptides (R11-Con. and p53C) was detected in T24 cells by fluorescence microscope. **C–D.** Inhibiting the proliferation of tumor cells expressing mutant p53 (i.e., T24) or wild-type p53 (i.e., 253J). Cell viability was determined by MTT assay. Results represent three different experiments, each of which was performed in six repeats.

The R11-p53C (R11-GG-GSRAHSSHLKSKKGQ STSRHKK-FITC) peptides by conjugating R11 with FITC-tagged p53C as the C-terminus separated by two glycines was synthesized, and the uptake efficiency was determined in UCB cells (i.e., T24) based on the fluorescence intensity of FITC conjugated with each peptide. A dose-dependent increase in fluorescence intensity was observed in R11-p53C and R11-Con (R11-GG-GSRAHSSHLESAEGQSTSRHKK-FITC) peptides; in contrast, p53C (GSRAHSSHLKSKKGQSTSRHKK-FITC) peptides had lower fluorescent intensity (Figure 1A). To visualize the intracellular localization of the peptides, fluorescence microscopy was applied in T24 cells after treating with three peptides, and the data indicated that majority of peptides were localized in the cytosol (Figure 1B). Consistent with the uptake efficiency results, R11-conjugated peptides exhibited a higher fluorescent intensity than others in T24 cells.

Subsequently, we examined the ability of growth inhibition induced by R11-p53C in T24 (mutant *p53*), 253J (wt *p53*) and SV-HUC (immortalized normal human bladder epithelial cell, wt *p53*) using MTT assay. Consistent with the recent report using p53 C-terminal peptide in prostate cancer cells [12], we found that R11-p53C inhibited cell proliferation in T24 cells (Figure 1C). Interestingly, it also exhibited similar effect in 253J cells regardless of the status of *p53* in a dose-dependent manner (Figure 1D). In contrast, R11-p53C peptide did not significantly affect the growth of SV-HUC cells (Supplementary Figure S1).

### Induction of p53 activities by R11-p53C peptide

**Figure 2:**
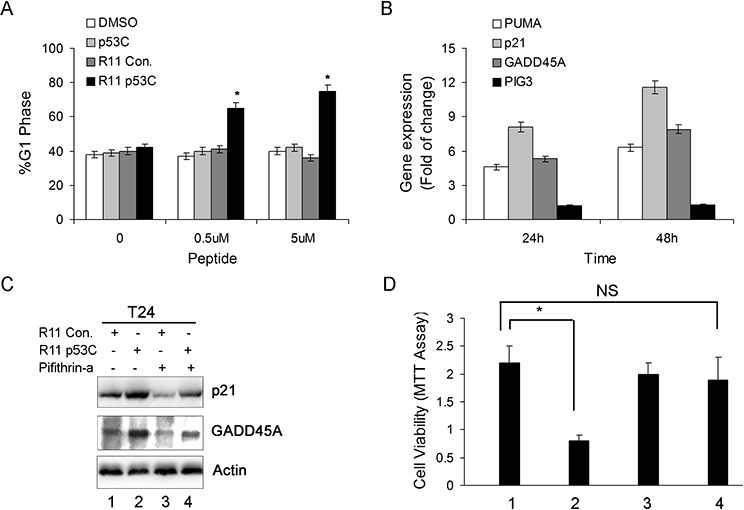
The effect of R11-p53C on p53-regulated gene expression and growth inhibition **A.** Dose-dependent induction of G1 cell cycle arrest by R11-p53C in T24 cells. Results represent three different experiments, each of which was performed in triplicates. *P < 0.05. **B.** Total cellular RNA was collected from T24 cells exposed to 5 μM R11-p53C or R11-Con. then subjected to qRT-PCR for determining PUMA, p21, GADD45A and PIG3 at the indicated time. **C.** T24 cells were pre-treated with pifithrin-α 20 μM (or DMSO) before 5 μM R11-p53C or R11-Con treatment for 48 hrs then subjected to western blot analysis. **D.** T24 cells were pre-treated with pifithrin-α (20 μM) or DMSO before 5 μM R11-p53C or R11-Con treatment for 48 hrs then subjected to MTT assay. Data represent mean and standard deviation from three different experiments; each experiment was carried out in triplicates.

Next, we also found that R11-p53C peptide could induce a G1 cell cycle arrest in T24 cells (Figure 2A). These results are consistent with a previous report that certain cancer cells with *p53* mutations are susceptible to CPP-p53C peptide [13]. To further determine whether the inhibitory effect by R11-p53C peptide in T24 cells was dependent on p53 activities, we examined the expression of several p53-regulated genes by qRT-PCR. In T24 cells, R11-p53C peptide was able to induce the expression of p53 target genes, including *p21, PUMA* (*p53*-up-regulated modulator of apoptosis), and *GADD45A* (growth arrest and DNA damage-inducible 45a) but not *PIG3* (*p53* induced gene 3) (Figure 2B). These results indicate that R11-p53C peptide can increase p53 transcriptional function in UCB cells.

To further ascertain that R11-p53C exerts its effect through p53-mediated pathways, we tested the effect of pifithrin-α, a specific inhibitor of p53, on R11-p53C-induced target gene expression and growth inhibition (31). The expression of *p21* and *GADD45A* protein was examined by western blot in T24 cells treated with or without pifithrin-α. Pifithrin-α significantly impaired the expression of *p21* and *GADD45A*, suggesting that its mechanism controlling the expression of these genes is p53-dependent (Figure 2C). As expected, pifithrin-α could also antagonize R11-p53C-mediated growth inhibition in T24 cells (Figure 2D). Taken together, these data indicate that R11-p53C is able to activate p53-dependent pathway and plays an important role in growth inhibition of UCB cells.

### Systemic delivery of R11-p53C peptide inhibits orthotopic tumor growth

**Figure 3:**
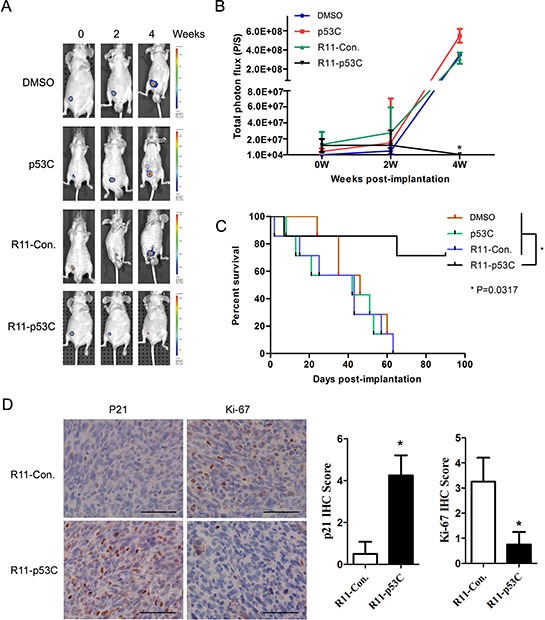
Systemic R11-p53C peptide administration inhibited orthotopical tumor growth **A–B.** T24 orthotopical tumor growth after R11-p53C treatment. T24-luc cells were instilled into the bladder to develop a superficial bladder cancer model (*n* = 6/group) at Day 0 and randomized to receive 200 μl of peptides and control twice a week and repeated 3 weeks. Tumor growth was determined. **C.** The survival curves were determined in every group. *P < 0.05. **D.** Representative pictures of p21 and Ki-67 IHC staining in T24 orthotopical xenograft tissues with or without R11-p53C treatment. The scale bar represents 100 μm. Quantification analyses of IHC score were shown (right panel).

We next assayed the ability of the R11-p53C peptide to alter the tumor burden and increase the longevity of mice bearing T24 orthotopic tumors. Tumor burden was monitored with *in vivo* imaging system (IVIS) in mice treated with R11-p53C peptide or the controls for 4 weeks (Figure 3A). R11-p53C peptide significantly decreased the tumor burden compared with the control peptides (Figure 3B). The animal survival curve showed that the p53C-treated mice rapidly succumbed to tumor burden with a mean survival time of 46 days (Figure 3C). Mice treated with control peptide (i.e., R11-Con.) succumbed to their tumor burden with similar mean survival time. In contrast, orthotopic bladder tumor-bearing mice treated with R11-p53C peptide survived much longer after drug injection (Figure 3C, *P* = 0.0317). Also, we observed a significant increase of p21 staining and decrease of Ki-67 staining in the xenograft tissues after R11-p53C peptide treatment (Figure 3D), indicating the *in vivo* anticancer effects of R11-p53C peptide is p53-dependent. These observations demonstrate the effectiveness of the small peptide in suppressing tumor growth and extending the survival of animals bearing UCB in a p53-dependent manner.

### Unique biodistribution of R11-conjuncted peptides

From the *in vivo* evaluation of the tissue distribution of three peptides (p53C, R11-Con. and R11-p53C) in nude mice bearing T24 orthotopic tumor, we also showed that R11-conjuncted peptides exhibited a specific uptake in bladder or orthotopic bladder tumors tissues 2 hrs after intraperitoneal injection, because the peptide uptake was much lower in other organs (Supplementary Figure S2).

### R11-p53C peptide inhibits the growth of lung metastatic tumor

**Figure 4:**
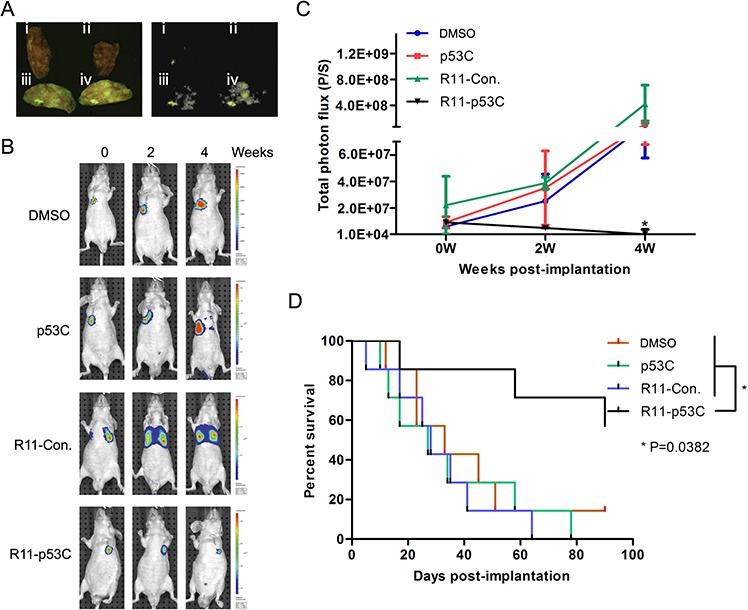
Systemic R11-p53C peptide administration inhibited T24 lung metastatic tumor growth **A.** R11-p53C peptide can be specifically detected in the lung metastatic lesions. Nude mice were intravenously injected with T24 cells for 14 days (*n* = 6/group). Tissues were collected 30 mins after R11-p53C and control peptides injected. I DMSO, II p53C, III R11-Con, IV R11-p53C. **B–C.** The BLI intensity was serially monitored (B) and quantified (C) in every group. **D.** The long-term survival of mice harboring lung metastatic burden after R11-p53C/control peptides/DMSO treatment.

In order to further show the specific uptake and broaden the application of R11-p53C in the metastatic tumors *in vivo*, we developed the lung metastatic animal model by injecting T24-luc cells into the tail vein. The lung metastasis model was observed by fluorescence microscopy. As expected, fluorescence-labeled R11-p53C and R11-Con. peptides could be readily detected in metastatic tumor lesions but not normal lung tissue (Figure 4A), indicating the tissue specificity of R11-conjuncted peptides in UCB. Early indications of metastasis were observed within 14 days after T24-luc cell injection. The initial signals were confirmed by sequential imaging (Figure 4B and 4C). Almost all lung metastatic lesions showed a gradual decrease in BLI intensity over time in R11-p53C treated group. Further, mice bearing metastatic bladder cancer succumbed to tumor burden when treated with DMSO, p53C or R11-Con., with a mean survival time of 33 days, 28 days and 27 days after tumor cell injection, respectively (Figure 4D). In contrast, R11-p53C peptide treatment could significantly increase animal survival rate, because 80% animal survived more than 60 days. Taken together, these observations demonstrate that R11-p53C could significantly decrease metastatic tumor burden and increase the overall survival.

Despite of these encouraging observations, R11-p53C peptide-treated animals still have some tumor burden, which could be due to the emergence of peptide resistant tumor cells or insufficient drug dose. To distinguish these possibilities, we isolated T24-luc cells recovered from tumors after 4 weeks of peptide treatment. T24 cells treated with R11-p53C peptide exhibited similar G1 cell cycle arrest as parental cells (Supplementary Figure S3). This observation rules out the possible acquisition of resistance in T24 cells. Thus, more treatment schedule is expected in order to achieve complete eradication of cancer cells.

### *In vivo* toxicity of R11-p53C peptide

**Figure 5:**
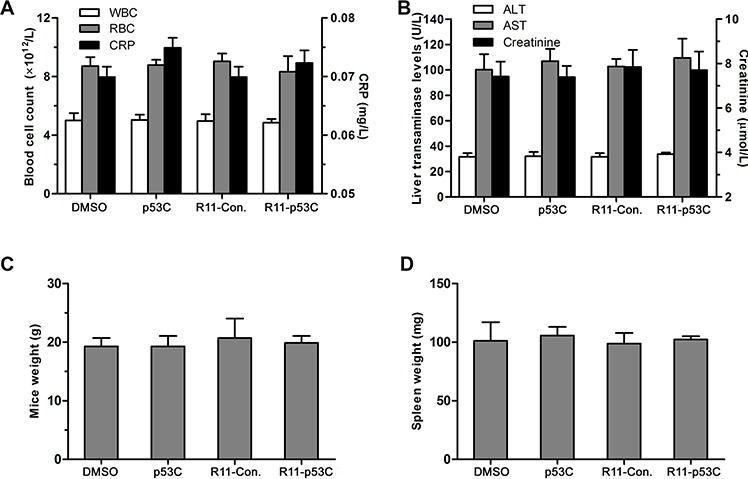
*In vivo* toxicity of R11-p53C peptide **A.** Levels of RBC, WBC and CRP from mice injected with R11-p53C or control peptides pretreated 24 hrs by intraperitoneal injection. **B.** Serum levels of AST, ALT and creatinine from mice injected with R11-p53C (20 mg/kg) or control peptides pretreated 72 hrs by intraperitoneal injection. **C.** Mice weights were measured at 3 days after injected with R11-p53C or control peptides. **D.** Spleen weights were measured from mice injected with R11-p53C or control peptides treated for 3 weeks. Means ±SD shown (*n* ≥ 3). Background levels were 43 ± 14.3 U/l for AST, and 29 ± 4.2 U/l for ALT. ALT, alanine aminotransferase; AST, aspartate aminotransferase; WBC, white blood cell; RBC, red blood cell; CRP, C-reactive protein.

After confirming the potency of R11-p53C in inhibiting UCB *in vivo*, we next sought to evaluate whether this compound had adverse side effects in mice when applied systematically. As shown in Figure 5A, the number of red blood cells, white blood cells and the level of C-reactive protein remained unchanged after 24 hrs of treatment with peptide, indicating minimal inflammatory responses toward these peptides [14]. Accordingly, liver transaminase levels (i.e., ALT/AST) remained the same in control-treated mice and R11-p53C-treated mice after 72 hrs of treatment. In agreement with these observations, serum creatinine level remained unaffected among these groups (Figure 5B). An important parameter for clinical application of *in vivo* delivery of R11-p53C is to avoid triggering acute toxicity to the total body weight [15]. Our data indicated that R11-p53C or its control (R11-Con.) used for 3 days had no any adverse effects on body weight of T24-injected mice (Figure 5C). Furthermore, there was no significant decrease of the spleen weights after 3 weeks of treatment with peptides (Figure 5D). Together, these results indicate that the R11-p53C peptide does not induce significant acute or chronic hemolytic reaction.

## DISCUSSION

In the clinic, advanced-stage or disseminated metastatic UCB are often resistant to current chemotherapy, and new strategies for treating the disease are clearly needed. It's known that the dysfunction of p53 has been related to progression, prognosis and therapeutic response of tumors [16]. These observations led to the hypothesis that introduction of functional p53 protein will be a useful means to treat UCB. In this study, we demonstrate that small peptide derived from the p53 C-terminus can be effectively delivered via R11-mediated transduction and specifically modulate tumor biology *in vitro* and *in vivo*.

A number of studies have attempted to evaluate the potential of p53-related proteins or peptides delivery in cancer therapy. Although the efficient antitumor activity of the compounds has been confirmed, their utility is limited by some problems, such as cytotoxicity and lack of systemic biodistribution to disseminated metastatic sites [17]. In contrast, we observed that R11-conjuncted peptides exhibited a specific and efficient uptake by UCB cell lines and animal models without significantly cytotoxic effects on normal tissues in this study.

Mechanistically, other laboratories have shown that the growth inhibition of tumor cells by C-terminal p53-derived peptide was directly correlated with levels of mutant *p53* without toxicity to normal or tumor cells with wt *p53* [12, 18, 19]. However, the present result showed that the growth inhibition in bladder cancer induced by R11-p53C was both in mutant and wt *p53* while had no significant effect on normal cells. Furthermore, we found the transcription levels of several *p53* target genes were upregulated significantly after R11-p53C treatment. However, the induction was abolished in the present of pifithrin-α treatment. This provide us evidence that the anticancer efficacy of R11-p53C was truly achieved by activation or restoration of the of endogenous p53 protein, although the detail of the molecular mechanism is unclear.

Most studies on anticancer transduction peptides employed subcutaneous tumor model to determine the therapeutic efficacy [20, 21]; this model may not mimic cancer development particularly the influence of tumor microenvironment. In this study, the anticancer efficacy of R11-p53C was demonstrated by using orthotopic and metastatic UCB animal models. We proved that R11-p53C significantly decreased orthotopic and even distant metastatic tumor burden.

One of the most interesting findings in the present study is that delivery of the p53C peptide into *p53* mutated tumor cells not only inhibits bladder tumor growth *in vivo*, but also dramatically extends animal survival in orthotopic and lung metastatic cancer models. It's known that most patients succumb to cancer due to the metastatic disease [22]. Moreover, the ability to alleviate pathology and extend survival, not simply to reduce tumor volume, is the key to create a truly successful anticancer therapeutics in the clinic. Therefore, the present results suggest that R11-p53C peptide may become a potential clinical therapy option for aggressive UCB.

Combination chemotherapy has been the standard of care for metastatic UCB since late 1980s. However, up to 50% of metastatic patients are ineligible for this regimen due to the impaired renal function, co-morbidity preventing high-volume hydration, or severe acute toxicity (SAT) [23–25]. Our data indicated that R11-p53C transduction does not induce any significant cytotoxicity and hence this may be able to decrease the side effects in cisplatin-based combination chemotherapy.

In conclusion, we successfully developed a CPP, R11-p53C, which was specifically delivered and inhibited UCB. Moreover, this study demonstrated that R11-p53C induced a significant inhibition of tumor growth not only regardless the state of *p53*, but also in orthotopic and distant metastasis tumor animal models with an activation or restoration of p53-dependent manner. We feel confident that the peptide could be a promising therapeutic drug for the treatment of UCB, especially be significant for metastatic patients.

## MATERIALS AND METHODS

### Peptide synthesis

p53C (GSRAHSSHLKSKKGQSTSRHKK-FITC), R11-p53C (R11-GG-GSRAHSSHLKSKKGQSTSRHKK-FITC) and R11-Con. (a mutant form of R11-p53C: R11-GG-GSRAHSSHLESAEGQSTSRHKK—FITC) peptides were synthesized by automated peptide synthesizer using the standard solid-phase chemistry. All peptides were purified to > 95% purity by reverse-phase high-performance liquid chromatography. The structure of each synthesized peptides was confirmed by mass spectrometry. Peptide stocks (10 mM) were prepared in DMSO and stored in aliquots at −20°C.

### Cell culture

The human UBC cell lines (T24 and 253J) and the immortalized bladder epithelial cell line SV-HUC were obtained from American Type Culture Collection (ATCC, Manassas, VA) and maintained in DMEM (Invitrogen, Carlsbad, CA) supplemented with 5% fetal bovine serum in a humidified incubator at 37°C with 5% CO_2_. T24-luc sublines were generated after stable transfection with the luciferase gene vector and G418 selection as our previous reports [26] and maintained in DMEM supplemented with 5% fetal bovine serum.

### Cellular uptake and subcellular localization *in vitro*

To determine the uptake efficiency of peptides, 1 × 10^5^ cells per well were plated in a 12-well plate. Next day, different concentrations of FITC-tagged peptides were incubated with cells for 30 mins. The total cell number was determined and cells were lysed in Tris [50 mM Tris-HCl (pH 7.5), 150 mM NaCl, 5 mM EDTA, 1% Triton X-100]. The fluorescence intensity was examined by fluorometer (excitation 490–500 nm; emission 515–525 nm). To determine the subcellular localization of peptides, cells were treated with three peptides for 30 mins and fixed with 4% paraformaldehyde in PBS plus 4′,6-diamidino-2-phenylindole (DAPI, 1 μg/mL; Sigma, St. Louis, MO) counterstaining, and then examined under fluorescence microscope.

### Cytotoxicity and cell cycle assay

To assess the cytotoxicity of peptides, 1 × 10^3^ cells were seeded in a 96-well plate for 24 hrs and then washed once with serum-free media before peptides treatment in a final volume of 100μl in DMEM containing 2% FBS. After incubation at 37°C for 1 hr, cells were washed twice with serum-free media and replaced with fresh DMEM containing 2% FBS. Fresh peptide was added daily for 3 days. The cell viability was determined at the indicated time after the initial treatment using 3-(4, 5-dimethylthiazol-2-yl)-2, 5-diphenyltetrazolium bromide (MTT) assay (Roche, Indianapolis, IN) following manufacturer suggested protocol.

For cell cycle analysis, cells were stained with 10 ng/ml propidium iodide after peptides treatment for 24 hrs and then subjected to FACS analyzed by CellQuest software (Becton Dickinson, Palo Alto, CA).

### RNA isolation and qPCR analyses

T24 cells seeded in 6-well plates were incubated with 5 μM R11-p53C or R11-Con. for 2 hrs. After washed, the cells were further incubated in fresh medium for 24 and 48 hrs, respectively. Total RNA was isolated using TRIzol reagent obtained from Invitrogen. The isolated RNA was reverse-transcribed using a kit from Invitrogen (One-Step PCR) primed by random oligo primers and then subjected to PCR analysis. Primers for *PUMA* (forward 5′-GGACGACCTCAACGCACAGT and reverse 5′-AATTGGGCTCCATCTCGGGG), *p21* (also known as *CDKN1A,* forward 5′-TTAGGGCTTCCT CTTGGAGAAGAT and reverse 5′-ATGTCAGAACCG GCTGGGGATGTC), *GADD45A* (forward 5′-AAGGGG CTGAGTGAGTTCAA and reverse 5′-TTTTCCTTCC TGCATGGTTC) and *PIG3* (also as known as *TP53I3,* forward 5′-CGGAATTCCGATGGGAGGGG AGCCG GGCC and reverse 5′-GGGGTACCCCCAGTTCACT CTTTATTTC) were used. All experiments were repeated at least thrice in duplicate.

### Western blot analyses

In brief, cell lysates were harvested, and proteins were separated by 10% SDS-PAGE and transferred to nitrocellulose membranes. Membranes were incubated with appropriate primary p21 (Cell Signaling Technology, Beverly, MA, USA) and GADD45A (Abcam, Cambridge, UK) antibodies overnight and horseradish peroxidase-conjugated secondary antibodies for 1 hr. Signals were detected using chemiluminescence (Pierce, Rockford, IL). Loading differences were normalized using a monoclonal Actin antibody.

### Effect of pifithrin-α on R11-p53C

For combination experiments, the T24 cells were seeded in 6 or 96-well plates overnight and pifithrin-α (20 μM) or DMSO was added 12 hrs before the peptide and every 12 hrs thereafter until analysis. The cells seeded in 6-well plates was isolated and analyzed using western blot analysis, and the proliferation of the cells seeded in 96-well plates were determined using a MTT assay as described above.

### *In vivo* tumor growth inhibition and biodistribution

Female nude (Balb/c nu/nu, 4–6 weeks age) mice were used for generating both orthotopic and metastatic models. The protocol is approved by Institutional Animal Care and Usage Committee of Xi'an Jiaotong University.

For the orthotopic model, intravesical tumor implantation of T24-luc cells was performed as previously described [26, 27]. Distant metastatic tumors were developed by intravenously injection of 0.5 × 10^6^ T24-luc cells in a final volume of 100 μL saline. The lung metastasis was allowed to develop 14 days post-injection and periodically imaged by bioluminescence imaging (BLI). To examine the effect of peptides on tumor growth, the mice were intraperitoneally injected with R11-p53C, or control peptides (20 mg/kg) in 200 μL saline twice a week for 4 weeks once the BLI signal in tumors was detectable. As a blank control, the mice were injected with saline containing DMSO. BLI was performed weekly to monitor the growth of tumor. The mice died before the end-point of experiment were autopsied immediately and the cause of the death was determined. If mice died before the end-point of experiment without any evidence of cancer, they were excluded from the data collection.

To determine the uptake efficiency of peptides in metastatic lesions, the groups of lung metastasis model were sacrificed 2 hrs after injection with peptides for determining biodistribution using fluorescence microscopy. For biodistribution, one group of animal (orthotopic tumor, *n* = 6) were intraperitoneally injected with peptides. Each organ from the group was harvested 2 hrs after injection and homogenized with Tris buffer and then the fluorescence intensity was examined by fluorometer. The uptake efficiency of peptides was determined by measuring the relative fluorescence intensity normalized to tissue weight from each organ.

### *In vivo* toxicity of R11-p53C peptide

In this experiment, animals (4–6 weeks age) were randomized into four groups (*n* = 6/group) and treated with peptides. Mice were weighted and sacrificed after treatment at the indicated times, tissues and blood were collected for analyses. Tissue sample analyses including spleen weight. Blood sample analyses including blood cell count and levels of C-reactive protein (CRP) were determined as previously reported [28]. Clinical chemistry parameters [alanine aminotransferase (ALT), aspartate aminotransferase (AST) and creatinine levels] of serum were analyzed by the Clinical Chemistry Laboratory.

### Immunohistochemical (IHC) staining

IHC was carried out with Dako Autostainer Plus system (Dako, Carpinteria, CA, USA) as described [29]. Briefly, sections were deparaffinized, rehydrated and subjected to antigen retrieval in citrate buffer (10 mM, pH 6.0) for 5 mins, and then endogenous peroxidase and alkaline phosphatase activity were blocked with Dual Block for 10 mins. The slides were then incubated overnight at 4°C with p21 (1:75 dilutions) and Ki-67 antibodies (1:100 dilutions). After washing, this was followed by incubation with EnVision secondary antibody for 30 mins at room temperature. Signals were detected by adding substrate hydrogen peroxide using diaminobenzidine (DAB) as a chromogen followed by hematoxylin counterstaining. Finally, the staining result was considered higher expression (intensity 2 or 3 and percent category 2 or 3) or lower expression (intensity 0 or 1, or more but percent category 0 or 1). For each section, the total score was calculated by multiplying the scores of intensity and percentage.

### Statistical analysis

All statistical analyses were performed using SPSS 15.0 (SPSS Inc., Chicago, IL, USA). Quantitative data are presented as mean ± SD, and the differences between two groups were compared by the 2-tailed Student's *t* test. Survival curves were plotted using Kaplan–Meier analysis. *P* ≤ 0.05 was considered statistically significant.

## SUPPLEMENTARY FIGURES


