# Supplementary material for: Inhibiting bladder tumor growth with a cell penetrating R11 peptide derived from the p53 C-terminus
Source: Oncotarget. 2015 Oct 8;6(35):37782–91. doi: 10.18632/oncotarget.5622 (PMC4741965; doi:10.18632/oncotarget.5622)
Supplement: Supplementary file 1 [file oncotarget-06-37782-s001.pdf]

SUPPLEMENTARY FIGURES

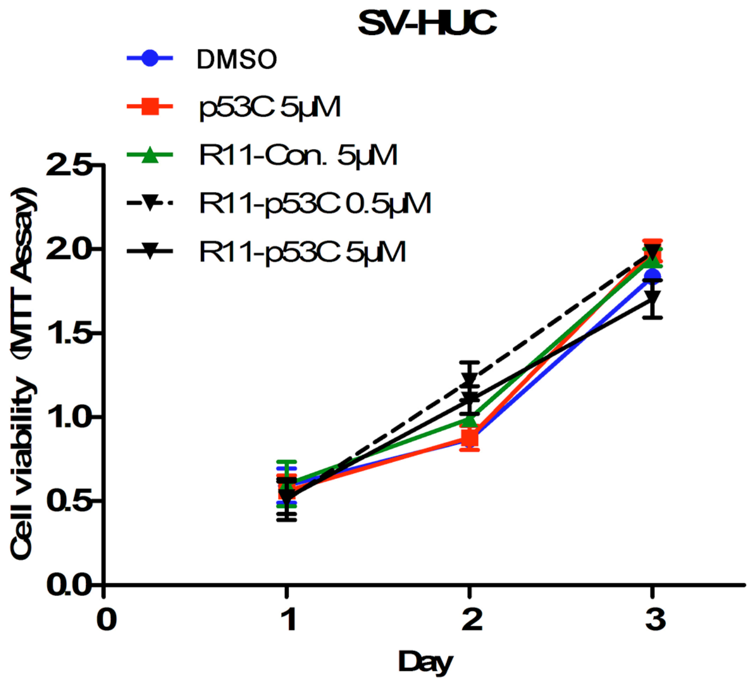

Supplementary Figure S1: The proliferation of SV-HUC cells expressing wild-type p53. Cell proliferation was determined by MTT assays. Results represent three different experiments, each of which was performed in six repeats.

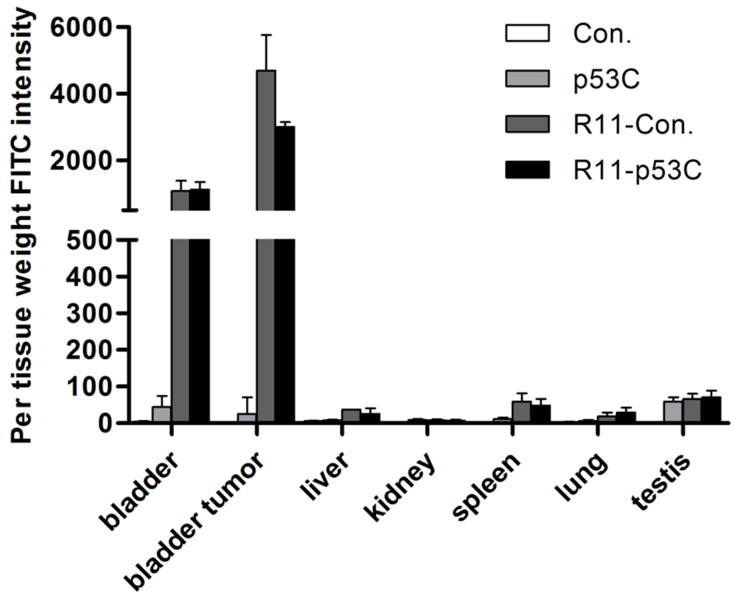

Supplementary Figure S2: Delivery of R11-p53C to orthotopic T24 tumors after IP administration to nude mice. The quantitative analysis of fluorescence from each tissue treated with R11-p53C peptide or control was carried out 2 hrs after injection and the fluorescence intensity was examined by fluorometer. The uptake efficiency was normalized to tissue weight from each organ.

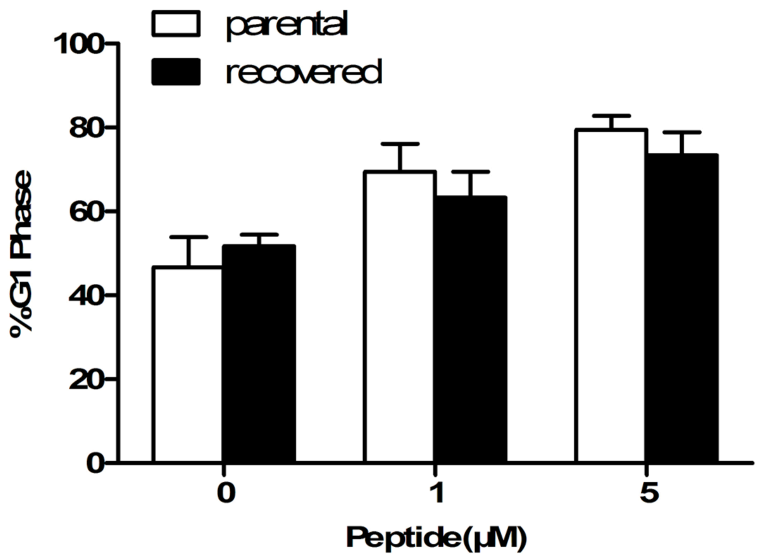

Supplementary Figure S3: No emergence of peptide-resistant tumor cells after systemic administration. T24 cells were recovered from nude mouse treated with R11-p53C peptide and grown in DMEM/10% FBS with 600 g/ml G418. Recovered cells were treated with increasing concentrations of R11-p53C peptide and then analyzed for DNA content by flow cytometry 24 hrs later.
